# Experimental study on in-situ simulation of rainfall-induced soil erosion in forest lands converted to cash crop areas in Dabie Mountains

**DOI:** 10.1371/journal.pone.0317889

**Published:** 2025-02-07

**Authors:** Gao Li, Tao Yang, Rui Chen, Haogang Dong, Feng Wu, Qinghua Zhan, Jinyan Huang, Minxuan Luo, Li Wang

**Affiliations:** 1 Changsha General Survey of Natural Resources Center, China Geological Survey, Changsha,; 2 Key Laboratory of Geological Hazards on Three Gorges Reservoir Area, Ministry of Education, China Three Gorges University, Yichang; Wallaga University, ETHIOPIA

## Abstract

Soil erosion is a pervasive global challenge and a significant ecological and environmental concern in China. Its occurrence frequently triggers ecological crises, including soil degradation and water contamination. It is of great scientific and practical significance to study the factors influencing the mechanism of soil erosion occurrence. Economic development in the Dabie Mountains of China has necessitated the conversion of vast tracts of forest land into economic crops, notably tea gardens and orchards, thereby disrupting soil structure and precipitating large-scale soil erosion. Rainfall serves as the primary catalyst for soil erosion in this region. Therefore, this study was designed to reveal the evolution characteristics of rainfall-induced slope erosion and the key influencing factors in the forest land converted to cash crop area in Dabie Mountains. It focused on a tea plantation slope of the Dabie Mountains, employing four rainfall scenarios, i.e. light rain, moderate rain, heavy rain, and heavy rain following drought, to conduct in-situ simulation experiments, mirroring the prevalent rainfall patterns in the study region. Monitoring stations for soil moisture content, slope runoff, and soil erosion were strategically positioned at varying depths across experimental plots with vegetation cover percentages of 20%, 40%, and 60%. Mathematical methods of descriptive statistics were used to analyze the monitored runoff, soil erosion and soil water content data, and to study the characteristics of their changes and response relationships. The findings underscore that rainfall prompts a swift surge in surface soil moisture, destabilizing the soil surface and culminating in slope erosion; thus, the rate of change in surface soil moisture content emerges as a pivotal indicator for predicting slope soil erosion. Furthermore, within the bounds of rainfall infiltration, preceding drought conditions followed by intense rainfall exacerbate soil erosion accumulation, highlighting the significance of initial soil moisture content as a critical factor. Lastly, for the economic crop cultivation zones in the Dabie Mountains, achieving a vegetation cover of 40% or more can significantly enhance soil water retention capacity and the overall soil and water conservation efficacy.

## 1. Introduction

Soil erosion refers to the whole process of destruction, denudation, transit and deposition of soil, soil matrix and other ground constituents under the action of external forces such as water power and wind power. Soil erosion often triggers a series of problems such as the decline of soil fertility, the weakening of land productivity, and the deterioration of the ecological environment [1,2]. Soil erosion, a pressing ecological and environmental issue of global concern, poses a formidable hindrance to sustainable socio-economic development. According to research [3,4], more than 65% of the world’s land has been subjected to land degradation in varying degrees, with soil erosion and desertification occurring as the main forms. The global soil erosion area has reached 25 million km^2^, accounting for 18.5% of the total land area, and the amount of soil erosion generated annually is as high as 60 billion tonnes. In recent years, economic losses due to soil erosion problems have severely constrained China’s development [5,6]. Recent findings from the dynamic monitoring of national soil and water erosion in 2022 reveal that China has a total area of 2,653,400 km^2^ of soil and water erosion, of which 936,100 km^2^ is moderately or severely eroded, accounting for 35.28% of the total area of soil and water erosion in China [7]. Notably, the densely inhabited areas in Dabie Mountains confront the dual challenges of a fragile ecological environment and economic hardship. In pursuit of economic development, the mountainous region has transformed extensive areas of forest land into tea plantations, orchards, and other cash crops, leading to the degradation of soil structure and subsequently, the emergence of large-scale soil erosion [8]. So far, the total area of soil erosion in the Dabie Mountains has reached 9520 km^2^, with an average annual erosion modulus reaching an alarming 2864 t/km^2^ [9]. Therefore, it is urgent to carry out in-depth research on the evolution mechanism of soil erosion and soil and water conservation measures in this region. Up to now, only some researchers have carried out exploratory work on soil erosion in the Dabie Mountains, mainly through remote sensing to monitor the relationship between the amount of soil erosion in the Dabie Mountains and the changes in land-use types [10,11]. However, many years have passed since the research work was carried out, and the vegetation types, vegetation cover and soil erosion in the Dabie Mountains area have changed considerably. Then, the important influencing factors of soil erosion and research methods still need further in-depth study. Over the past century, scholars both domestically and internationally have conducted extensive research on the factors influencing soil erosion and its evolutionary processes [12–14].

Ellison [15] first elucidated the correlation between soil erosion and rainfall intensity, highlighting that in the initial stages of rainfall, greater intensity leads to more severe splash erosion. However, as a crust forms on the slope’s soil surface, runoff increases while splash erosion significantly diminishes. As research progresses and methodologies evolve, experts and scholars have embraced monitoring and experimental tools to quantitatively analyze the impact of various factors on slope erosion. In soil erosion monitoring systems, remote sensing stands out due to its swiftness and timeliness [16,17]. Since 2019, with the widespread adoption of high-resolution (≤2 m) remote sensing imagery for dynamic erosion monitoring, scholars have utilized such data with the Chinese Soil Loss Equation (CSLE) to study soil erosion in the Yellow River alluvial area of Huaihe Basin and the Yellow River alluvial area of Northwest Shandong, yielding fruitful outcomes [18–20]. Nevertheless, remote sensing has limitations in unraveling the intricate mechanisms of soil erosion and its water-soil interaction impacts [21,22]. Thus, erosion simulation experiments have emerged as a pivotal method for scholars to delve into the mechanisms and influencing factors of erosion [23–26]. They have conducted indoor rainfall tests to investigate the effects of rainfall characteristics [27], topographic features [28], soil properties [29], and vegetation coverage [30] on slope erosion. While indoor model tests offer a convenient and expeditious means to study the evolution law and influencing factors of soil erosion, they have limitations as the test soil is often reconstructed, making it challenging to replicate real-world boundary conditions and environments [31–33]. Field monitoring, on the other hand, observes soil erosion dynamics through the establishment of standardized runoff plots. It employs long-term series to monitor multiple natural rainfall events, analyzing erosion’s response to rainfall intensity and type, as well as the correlation between sediment yield and rainfall factors [34–36]. This provides a theoretical foundation for erosion prediction and control measures. However, in exploring erosion’s evolution mechanisms, studies tend to focus solely on rainfall patterns, slope length and gradient changes, and runoff and sediment dynamics [37,38], neglecting the integration of rainfall infiltration to understand erosion’s response to soil moisture’s spatio-temporal variations or derived indicators.

Scholars have used many methods and tools to research soil erosion and achieved fruitful results. However, there are shortcomings in those methods. Currently, while remote sensing monitoring of soil erosion offers convenience and speed, it fails to address the issue of soil erosion in the study area from the perspective of rainfall infiltration mechanisms [39]. The long-term observation method allows for the analysis of soil erosion change mechanisms and influencing factors by monitoring rainfall, runoff, and soil erosion data over recent years. However, the increasing frequency of extreme weather in recent times has made it challenging to capture the typical rainfall characteristics of the study area over the past few decades within a short span, thereby posing certain limitations on the scope of the study [40]. Indoor simulation tests of soil erosion effectively address the difficulty in obtaining rainfall characteristics of the study area. This method relies on the collection of typical rainfall characteristics over the years in the study area and directly simulates the evolution of soil erosion characteristics of the test subject under rainfall conditions through rainfall tests [41]. However, indoor tests typically involve transporting soil samples from the study area to an indoor testing site and then conducting experimental studies after remolding. This process alters the actual boundary conditions of the in-situ geotechnical material, such as densities, porosities, permeability, and inter-particle forces, resulting in a decrease in the reliability of test results [42]. The in-situ soil erosion test comprehensively addresses several critical issues, including atypical rainfall data in the study area, inadequate research on rainfall infiltration mechanisms, and deficiencies in the real boundary conditions of geotechnical materials [43]. Currently, due to the challenges and high costs associated with building the necessary experimental equipment, researchers have primarily applied this research tool in the field of geological disasters and have achieved several significant findings [44,45]. Relatively little work has been conducted on in-situ tests of soil erosion. Therefore, conducting in-situ soil erosion tests in the Dabie mountains area of China represents a reliable means to identify the primary types of rainfall and collect data on soil erosion in the study area. This approach will facilitate the revelation of the soil erosion mechanism in the study area and, furthermore, enable the identification of key influencing factors for soil erosion in the region by varying different test conditions.

Soil erosion is a prominent ecological and environmental problem in the Dabie mountains [9,46], and field investigations have found that slopes planted with cash crops in the study area are prone to soil erosion under different types of rainfall conditions. How to carry out the study of soil erosion in the study area through rainfall in situ tests? What are the important factors influencing the occurrence of soil erosion in the study area? In this study, a cash crop planting area in the Dabie Mountains was used to carry out in-situ experimental research on soil erosion under different rainfall patterns and investigate the relationship among slope runoff, soil loss, and the response of water content and rainfall, so as to find out the change rate of soil water content and the law of the initial water content affecting soil erosion. The research results will provide a basis and guiding advice for the prevention and control of soil erosion in the Dabie Mountains.

## 2. Overview of the study area

**Fig 1:**
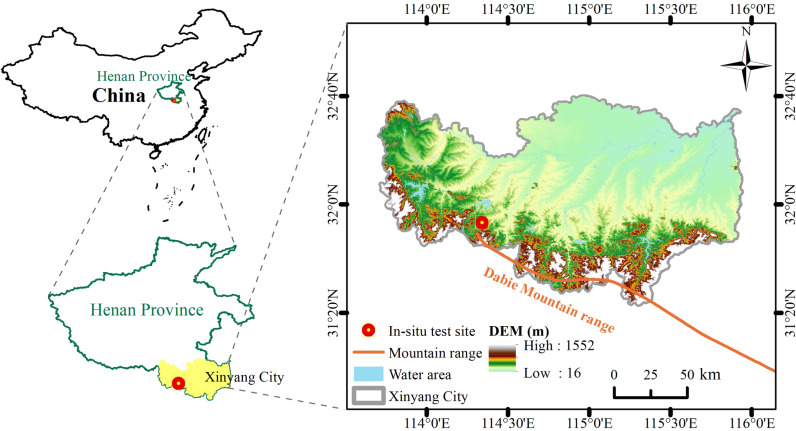
Geographic location of the study area. The geographic location of the study area and the digital elevation map of the Dabie Mountain region were prepared based on spatial data freely available from gis5g (http://gis5g.com/data/dxdm?id=34).

**Fig 2:**
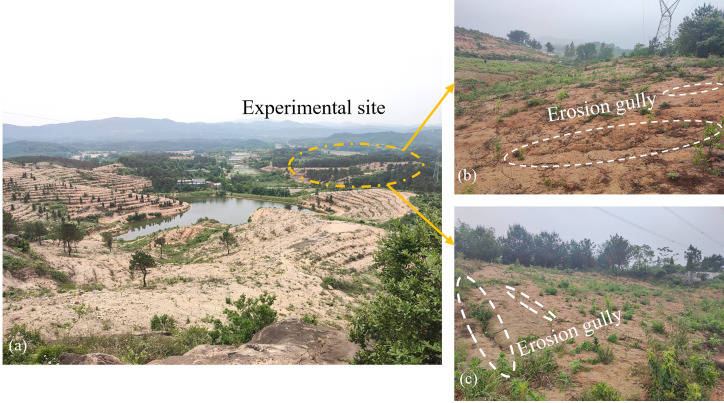
State quo of soil erosion in the study area: (a) Current status of soil erosion in the study area and location of the test site; (b) Erosion gully on the right side of the test site; (c) Erosion gully on the left side of the test site.

The present study site is located in the western section of the Dabie Mountains, and a sloping site planted with cash crops was selected in Xinyang City, Henan Province, to carry out rainfall-induced soil erosion in-situ experiments, and the geographic location of the study area is shown in Fig 1. The study area is a hilly mountainous terrain with steeper terrain, developed primary joints in rocks and thick weathering layer. The soil is relatively underdeveloped, primarily consisting of sandy soil and sandy loam, which exhibits poor cohesion and is prone to erosion. Affected by human activities, the vegetation in the study area has been partially cut down and planted with oil tea, tea and other cash crops instead (Fig 2). The overall vegetation coverage is low, and the overall soil erosion in the study area is strong-very strong, and the type of erosion is dominated by surface erosion and gully erosion, and the erosion is mainly caused by rainfall according to investigation.

## 3. Materials and methods

### 3.1. Overview of the experiment

A field artificial rainfall simulation system with simple operation, convenient transportation and good uniformity was designed for soil erosion in-situ test. The test system consists of four parts: the main body (nozzle, rainwater piping system), support frame, metering unit (water meter, rain gauge, etc.) and control unit (booster pump, water pressure gauge, flowmeter and frequency converter, etc.). As mentioned earlier, the test system designed in this study can overcome a number of critical problems such as atypical rainfall data and flaws in the real boundary conditions of geotechnical bodies. However, the in-situ test also has inherent limitations. Notably, it is significantly influenced by weather conditions, rendering it unsuitable for windy and rainy conditions, as this would compromise the rainfall simulation’s effectiveness. Furthermore, soil erosion is influenced by factors such as slope gradient, slope height, and soil characteristics. Consequently, compared to indoor simulation test systems, the in-situ test system lacks the capability to alter slope gradient, slope height, and soil type. It is noteworthy that the rainfall experimental system developed in this study possesses a broader applicability across various research objectives. For instance, different vegetation types can be cultivated in the study area to assess their respective soil and water conservation effects. This experimental setup can also simulate the impacts of recent extreme rainfall events on soil erosion, provided adequate water resources are available. More broadly, this test system can be utilized to conduct experimental investigations into rainfall-induced landslides.

This study was a trial funded by a grant from the author’s organisation, and the author, as the project leader of this grant, applied for a legal trial site from the local authorities in the early stage, and then constructed the trial site himself. During the trial work, only the author himself and relevant researchers were granted the access to the field, and therefore the author did not need a permit to enter the trial site. In the rainfall test, strict adherence to rainfall uniformity requirements is essential to guarantee the accuracy of test results. As illustrated in S1 Fig, based on the data collected from the nozzles, a targeted rainfall uniformity test was conducted. To minimize reading errors, cylinders of varying capacities were used to collect rainwater during the test. Specifically, a single row of hoses with multiple nozzles arranged at a spacing of 0.5–1 m was employed, as depicted in S2 Fig. Through iterative adjustments of the nozzle’s height above the ground, an optimal height of 1.8 m for the rainfall simulator was determined. Furthermore, adjustments were made to the nozzles to achieve a rainfall pattern resembling a fine mist. After 30 min. of testing, readings from the rain barrels, arranged from left to right, were recorded as 39 mm, 36 mm, 37.5 mm, and 42.5 mm, respectively. According to the *Sprinkler Irrigation Engineering Technical Specifications* (GB/T 50085-2007) [47], the rainfall uniformity coefficient (*C*_*u*_) was calculated. The test met the uniformity requirement when the *C*_*u*_ value was ≥  80%.

$$C_{u}=100(1-\frac{\Delta h}{h})$$ (1)

$$h=\frac{\sum_{i=1}^{\text{n}}h_{i}}{n}$$ (2)

$$\Delta h=\frac{\sum_{i=1}^{n} |h_{i}-\left.h\right|}{n}$$ (3)

**Fig 3:**
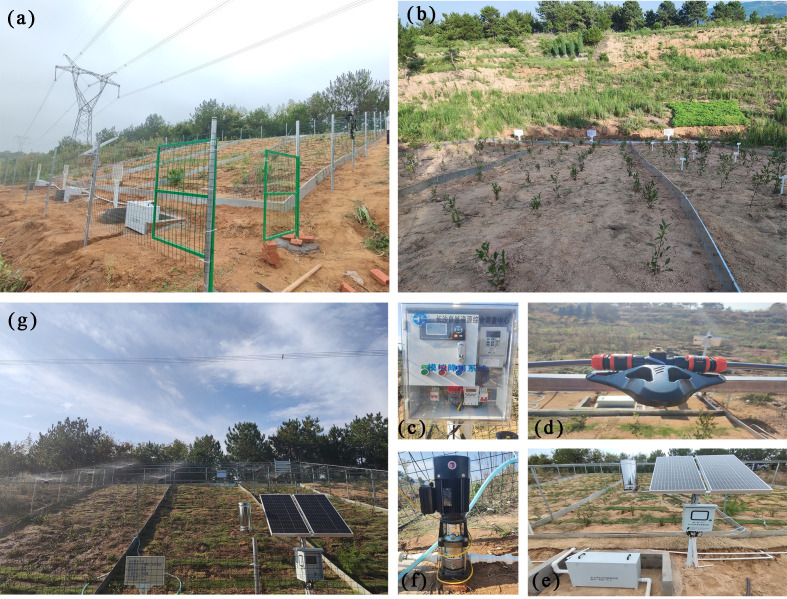
Diagram of the test site erection process: (a) Setting up the test site; (b) Tea planting; (c) Rainfall simulation test control system; (d) Rainfall nozzles; (e) Soil erosion collection system; (f) booster pump; (g) Rainfall uniformity test.

The rainfall uniformity coefficient *C*_*u*_ can be calculated by equations (1)–(3), where: *C*_*u*_ is the rainfall spray uniformity coefficient, in %; *�h* is the average height difference of spray water depth, in mm; *h* is the average spray depth, in mm; and *h*_*i*_ is the water depth at the measuring point, in mm. Substituting the test data into the above equations, the *C*_*u*_ is calculated to be 95.6% (>80%), indicating an acceptable rainfall uniformity. The test build process is shown in Fig 3.

**Fig 4:**
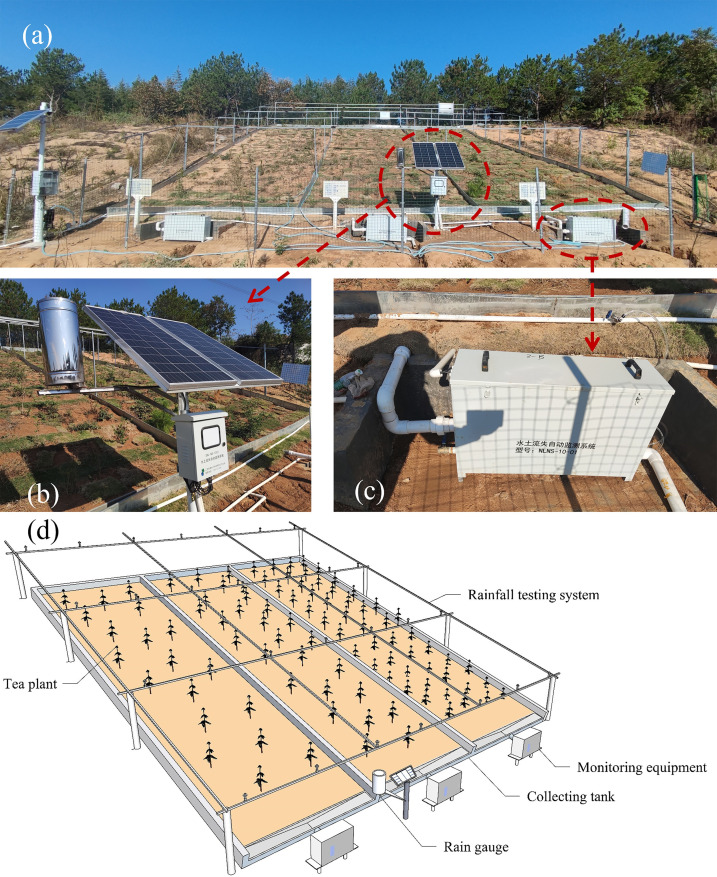
Rainfall in-situ test system on site: (a) rainfall in-situ test system on site; (b) rain gauge; (c) automatic soil erosion monitoring system; (d) schematic diagram of the rainfall in-situ test system.

The test monitoring system consists of monitoring sensors and data collectors that measure rainfall in real time through flow calculations and the deployment of rain gauges in the lower part of the test area. In this study, three adjacent test areas were designed and ECH2O-5 soil water content sensors were buried at different depths (10 cm, 20 cm, and 30 cm) in the three test areas. The runoff and soil loss were monitored using the NLNS-1-01 erosion monitoring system, and the data were integrated using the erosion monitoring and collection terminals. The test site plan and schematic diagram are shown in Fig 4, and the test flow is shown in S3 Fig.

### 3.2 Test plots and test program

The three neighboring test plots circled for this study were numbered 1–3# in sequence. The single test plot was 5 m long and 18 m wide, covering an area of 90 m^2^, and the average slope in the experiment was 15°. The test plots were planted with tea and the vegetation coverage was 20%, 40% and 60% in that order.

Rainfall in the Dabie Mountains is concentrated in summer, and soil erosion is mainly caused by rainfall. According to the local rainfall data of many years, the overall rainfall in the Dabie Mountains is uneven, with light rain, moderate rain, heavy rain occurring, and the multi-year average of the maximum single-day rainfall is about 40 mm[9,46] and droughts are often followed by heavy rainfall phenomena. Based on the local multi-year rainfall characteristics, four rainfall scenarios were designed, as shown in Table 1. For the light rainfall scenario, the rainfall duration was 50 min., with the overall test lasting a total of 150 min. For the medium rainfall scenario, the rainfall duration was 90 min., and the overall test duration amounted to 100 min. Both the first and second heavy rainfall scenarios featured a rainfall duration of 150 min., with an overall test duration of 180 min. for each. The fourth rainfall test had the same rainfall amount and duration as the third test, but the fourth rainfall test was preceded by a three-day exposure of the slope to simulate the occurrence of heavy rainfall after drought in the study area, and the actual intensity of rainfall was recorded by the rain gauge observation device during the rainfall period.

**Table 1:** Rainfall test program.

No.	Type of rainfall	Intensity of rainfall (mm/h)	Total rainfall (mm)	Total duration of the test (min)
1	light rain	9.9	8.3	150
2	Moderate rain	14.2	21.3	100
3	The first heavy rain	17.1	42.7	180
4	The second heavy rain	17.1	42.7	180

## 4. Results

Based on the rainfall p simulation program, this experiment primarily aimed to gather data on runoff volume, soil loss, and soil water content at various depths on the slope surface. It employed descriptive statistical methods to quantitatively analyze key data, including cumulative soil water content, cumulative runoff volume on the slope, runoff rate, soil loss volume, and soil loss rate, through the use of graphs and charts. Additionally, it aimed to further elucidate the relationships among the statistical data changes, thereby revealing the evolutionary characteristics and influencing factors of soil erosion in the study area.

### 4.1 Results of slope runoff in response to rainfall

**Fig 5:**
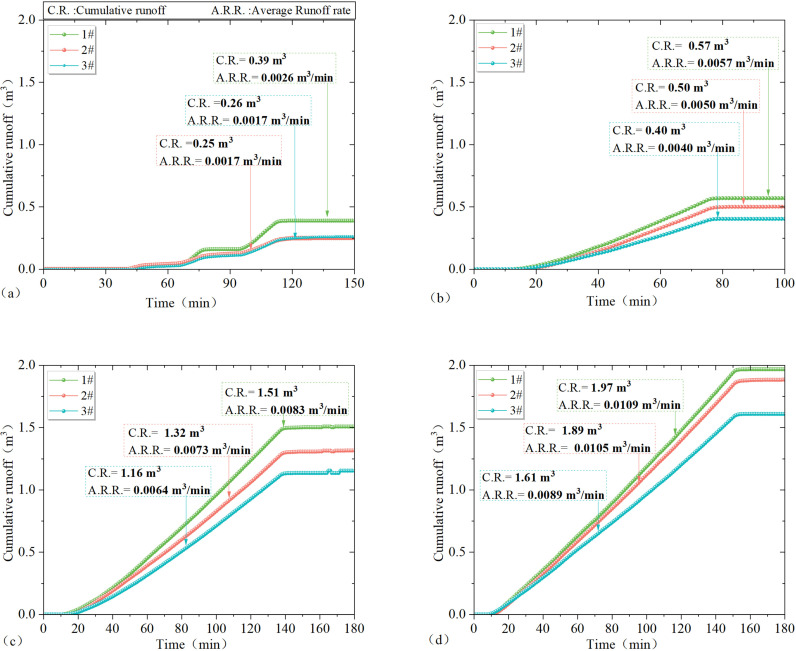
Results of slope runoff in response to rainfall: (a) cumulative meridional flow and runoff rates on the slopes of test sites 1–3# under light rainfall conditions; (b) cumulative meridional flow and runoff rates on the slopes of test sites 1–3# under moderate rainfall conditions; (c) cumulative meridional flow and runoff rates on the slopes of test sites 1–3# under the first heavy rainfall condition; (d) cumulative meridional flow and runoff rates on the slopes of test sites 1–3# under second heavy rainfall conditions.

From Fig 5(a)–(d), it can be seen that the slope runoff responded significantly to rainfall and the runoff volume and runoff velocity were different for different test areas under the same rainfall scenario. The cumulative runoff volume and average runoff velocity of test plots 1–3# under different rainfall scenarios in Fig 5(a)–(d) are included in Table 2.

**Table 2:** Cumulative runoff volume and average runoff rate on slopes under different rainfall scenarios.

	1#		2#		3#	
	Cumulative runoff (m^3^)	Average runoff rate (m^3^/min)	Cumulative runoff (m^3^)	Average runoff rate (m^3^/min)	Cumulative runoff (m^3^)	Average runoff rate (m^3^/min)
Light rain	0.39	0.0026	0.25	0.0017	0.26	0.0017
Moderate rain	0.57	0.0057	0.50	0.0050	0.40	0.0040
The first heavy rain	1.51	0.0083	1.32	0.0073	1.16	0.0064
The second heavy rain	1.97	0.0109	1.89	0.0105	1.61	0.0089

As illustrated in Fig 5(a)–(d), it is evident that, for the identical rainfall scenario, an increase in vegetation cover by 20% in test areas 1–3# results in an average reduction of at least 0.12 m^3^ in slope cumulative runoff volume and an average decrease of at least 0.00075 m^3^/min. in slope average runoff rate. For the same test areas, an increase in rainfall by 20 mm leads to an average increase of at least 0.5 m^3^ in cumulative runoff volume on slopes and an average increase of at least 0.0027 m^3^/min. in slope runoff rate.

### 4.2 Results of soil loss in response to rainfall

**Fig 6:**
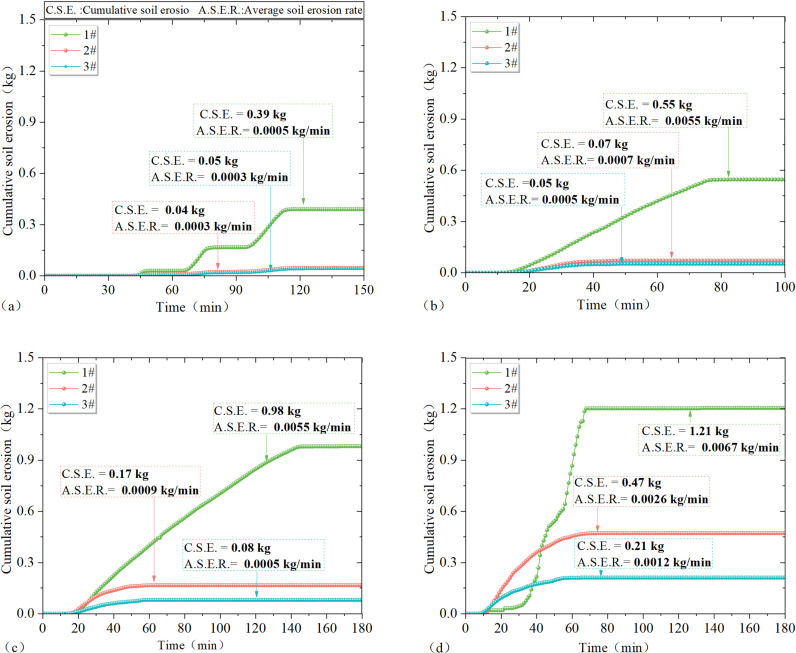
Results of soil erosion in response to rainfall: (a) cumulative soil loss and rate of soil loss from test sites 1–3# under light rainfall conditions; (b) Cumulative soil loss and rate of soil loss from test sites 1–3# under moderate rainfall conditions; (c) Cumulative soil loss and rate of soil loss from test sites 1–3# under the first heavy rainfall condition; (d) Cumulative soil loss and rate of soil loss from test sites 1–3# under the second heavy rainfall condition.

As seen in Fig 6(a)–(d), soil loss responded significantly to rainfall. Under the same rainfall scenario, the amount of soil loss varied in different test plots. The cumulative amount of soil loss and the average rate of soil loss under different rainfall scenarios for test plots 1–3# in Fig 6(a)–(d) are included in Table 3.

**Table 3:** Soil erosion accumulation and average soil loss rate under different rainfall scenarios.

	1#		2#		3#	
	Cumulative soil erosion (kg)	Average rate of soil erosion (kg/min)	Cumulative soil erosion (kg)	Average rate of soil erosion (kg/min)	Cumulative soil erosion (kg)	Average rate of soil erosion (kg/min)
Light rain	0.39	0.0005	0.05	0.0003	0.04	0.0003
moderate rain	0.55	0.0055	0.07	0.0007	0.05	0.0005
The first heavy rain	0.98	0.0055	0.17	0.0009	0.08	0.0005
The second heavy rain	1.21	0.0067	0.47	0.0026	0.21	0.0012

As illustrated in Fig 6(a)–(d), it is evident that under the same rainfall scenario, an increase in vegetation cover by 20% in test areas 1–3# leads to a reduction in average cumulative soil loss from slopes by at least 0.34 kg, and the average rate of soil loss decreases by at least 0.002 kg/min. With an increase in rainfall, the cumulative soil loss difference among the test areas increases. Specifically, for the same test area, an increase in rainfall by 20 mm results in an increase in cumulative soil loss on the slope by at least 0.15 kg and an increase in the average rate of soil loss on the slope by at least 0.0014 kg/min. Overall, under the same conditions, the average rate of soil loss accelerates significantly when rainfall increases from light to medium or heavier, while it decreases significantly when vegetation cover reaches 40% or more. Notably, a significant decrease in the average rate of soil loss is observed at 40% or higher vegetation cover.

**Fig 7:**
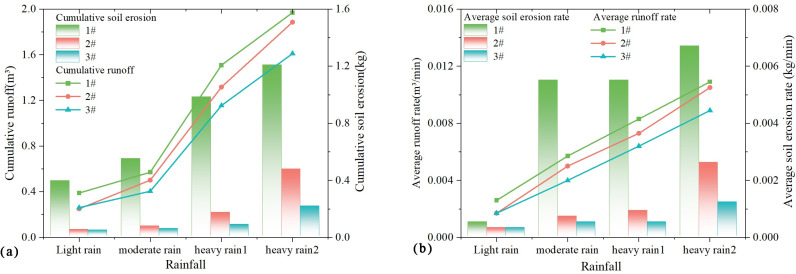
(a) Variation of soil erosion accumulation with rainfall; (b) Variation of average rate of soil erosion with rainfall.

From the results of the above experimental analyses, it can be seen that the response of slope erosion to rainfall is more obvious, and the results of the cumulative amount of erosion and the rate of erosion with rainfall are plotted in Fig 7.

Combining the results of the above analyses and Fig 7, it can be concluded that rainfall is an important influence factor on slope erosion, and under the same conditions, the cumulative amount of slope erosion increases with the increase of rainfall. For rainfall up to 40mm or more, slope runoff can be up to 0.94m^3^ and soil loss can be up to 0.43 kg. Under the same conditions, the cumulative amount of soil erosion on slopes decreases with the increase of vegetation coverage. When the vegetation cover reaches 40% and above, the slope runoff can be reduced by 0.14 m^3^ and the soil loss can be reduced by 0.48 kg, so the soil and water conservation effect of vegetation is remarkable.

From the results of the two heavy rainfall tests, it can be seen that the accumulation and rate of change of soil erosion on the slope surface during the second rainfall differed significantly from the results of the first rainfall test. The reason is that the test soil was exposed to sunlight prior to the second heavy rainfall test, resulting in a different initial soil moisture content between the two tests. The response of slope erosion to changes in soil moisture content at different depths is analyzed further below.

### 4.3 Analysis of slope erosion in response to changes in soil water content

**Fig 8:**
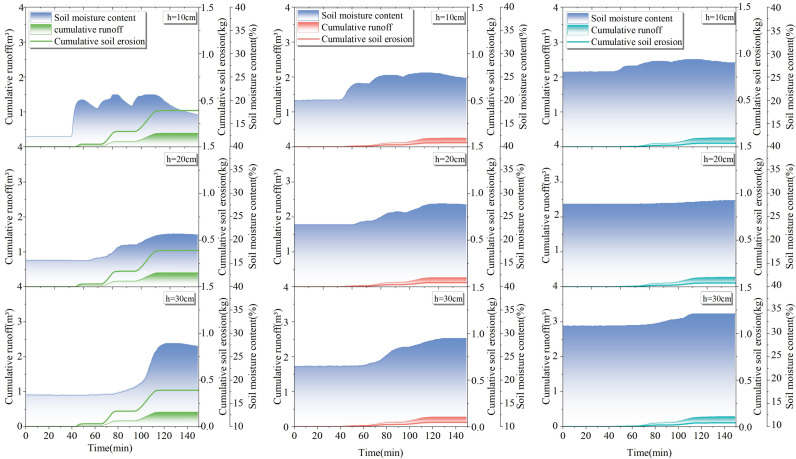
Response of soil erosion to soil water content at 10 –30 cm depth in test sites 1-3# under light rainfall conditions.

**Fig 9:**
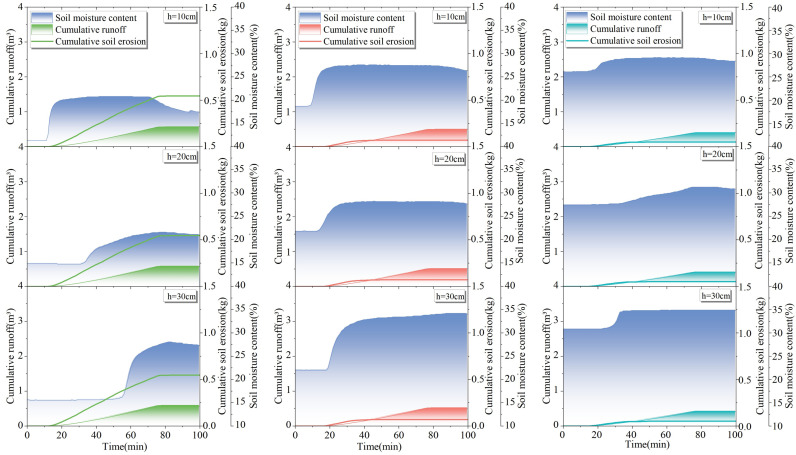
Response of soil erosion to soil water content at depths of 10 –30 cm in test sites 1–3# under moderate rainfall.

**Fig 10:**
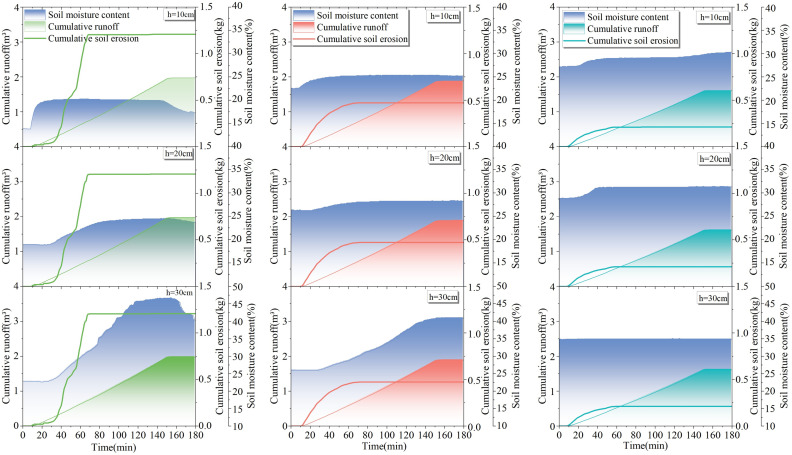
Response of soil erosion to soil moisture content at 10 –30 cm depth in test sites 1–3# under the first heavy rainfall conditions.

**Fig 11:**
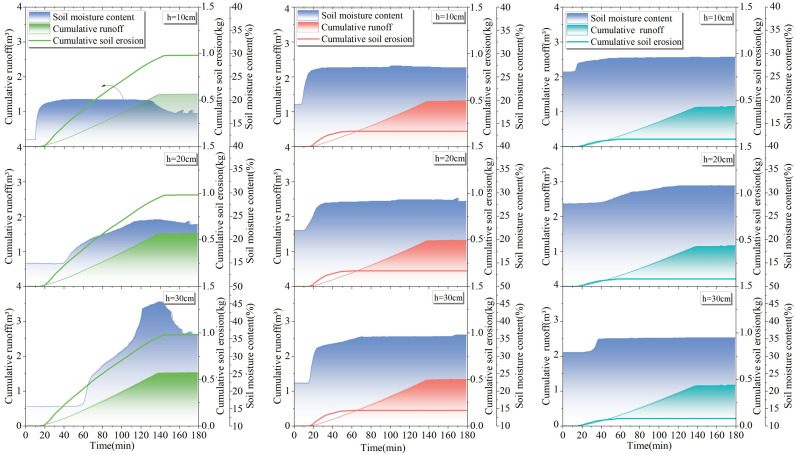
Response of soil erosion to soil water content at 10 –30 cm depth in test sites 1–3# under second heavy rainfall conditions.

Combined with Fig 8–11 and Table 4, it can be seen that with the increase of rainfall, the water content in 1–3# test plots at different depths increased differently; with the increase in depth, the change in soil water content showed a lag phenomenon, and the lag is related to the permeability of the soil. According to the change in moisture content, the average infiltration depth of the experimental soil at each position can reach 30 cm.

**Table 4:** Initial and final soil water content under different rainfall scenarios.

		1#		2#		3#	
Type of rainfall	Depth of soil	Initial soil moisture content (%)	Final soil moisture content (%)	Initial soil moisture content (%)	Final soil moisture content (%)	Initial soil moisture content (%)	Final soil moisture content (%)
Light rain	10 cm	12.2	16.9	20.0	24.7	26.1	28.0
	20 cm	15.6	21.0	23.2	27.5	27.6	28.4
	30 cm	16.8	27.1	22.8	28.8	31.5	34.1
Moderate rain	10 cm	11.4	17.4	18.7	26.3	26.2	28.4
	20 cm	14.8	21.0	21.7	27.6	27.4	30.8
	30 cm	15.6	27.2	22.0	34.0	30.7	34.8
The first heavy rain	10 cm	13.9	17.3	22.5	25.2	27.2	30.3
	20 cm	18.9	23.7	26.3	28.2	28.8	31.3
	30 cm	22.8	40.3	26.0	40.9	34.7	34.8
The second heavy rain	10 cm	11.5	17.15	19.1	27.0	26.1	29.3
	20 cm	14.9	23.18	21.9	28.2	27.6	31.5
	30 cm	15.5	36.16	22.2	35.9	30.8	35.1

Under the same rainfall scenario, for the same soil depth, the initial soil moisture content and final soil moisture content increased with the increase of vegetation coverage. The average increase in final soil water content at the same depth was 5.5% in 2# test plot compared to 1# test plot, and the average increase in final soil water content was 1.9% in 3# test plot compared to 2# test plot. The soil water-holding capacity increased significantly when the vegetation coverage was 40% or above, which is similar to the results discussed in the previous section.

As shown in Fig 8–11, the rainfall amount and duration of the two heavy rainfalls were the same, but the difference in slope erosion was significant. The results of the two heavy rainfall tests showed that the initial soil water content was different in each test plot before the two heavy rainfall tests, in which the second heavy rainfall reduced the initial soil water content by an average of 4.6% in 1# test plot, 3.9% in 2# test plot, and 2.1% in 3# test plot compared to the first heavy rainfall test. Following the two heavy rain tests, notable changes were recorded in the slope’s hydrological and erosional responses. In the 1# test plot, the total runoff volume increased by 0.49 m^3^, and soil loss escalated by 0.23 kg during the second heavy rain test. Similarly, in the 2# test plot, the total runoff augmented by 0.57 m^3^, accompanied by a 0.31 kg surge in soil loss. The 3# test plot witnessed a 0.46 m^3^ increase in total runoff and a 0.13 kg rise in soil loss. These findings underscore the significance of varying initial soil water content, as it influences the slope’s susceptibility to erosion under identical rainfall conditions. In essence, when intense rainfall occurs subsequent to drought conditions, keeping all other factors constant, the magnitude of soil erosion on slope undergoes a substantial augmentation.

**Fig 12:**
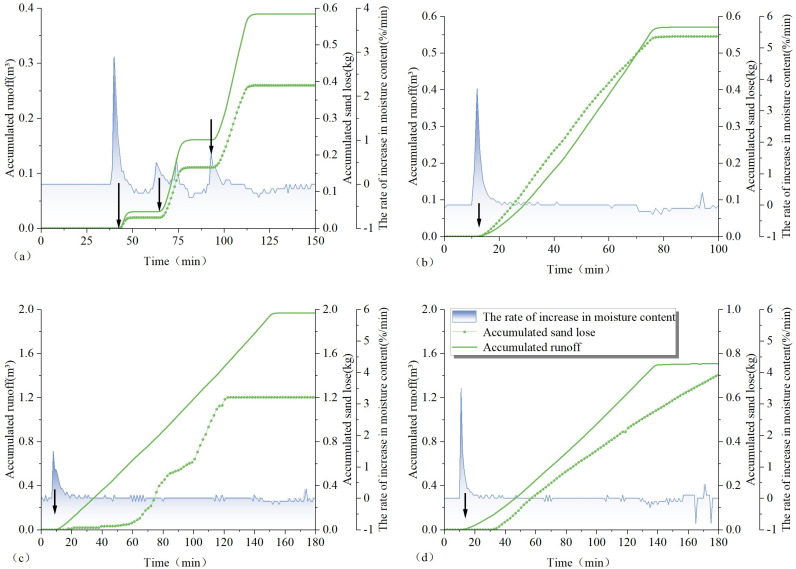
Response of slope erosion to the rate of change of soil water content in the 1# test plot: (a) response of soil erosion to the rate of change of soil water content at test site #1 under light rainfall conditions; (b) response of soil erosion to the rate of change of soil water content at test site 1# under moderate rainfall conditions; (c) response of soil erosion to the rate of change of soil water content at test site 1# under the first heavy rainfall condition; (d) response of soil erosion to the rate of change in soil water content at test site 1# under second heavy rainfall conditions.

Overall, slope erosion occurred when soil water content increased due to the increase of rainfall. Notably, a substantial increase in soil water content within the top 10 cm of the soil profile elicits a pronounced response in terms of slope soil erosion. The results of the slope erosion response to the rate of change in soil water content are plotted in Fig 12 for further analysis.

### 4.4 Analysis of the response of slope erosion to the rate of change in soil water content

For brevity, the slope runoff and soil erosion in the 1# test plot were selected for comparative analyses with the rate of change of surface soil water content. As seen in Fig 12, the rate of increase in soil water content under the light rainfall test conditions showed three high values, 2.9%/min., 0.5%/min., and 0.6%/min. at the 40th, 63rd, and 74th min., respectively, and the slope runoff and soil erosion also started three times. Among them, the starting time of slope runoff was basically the same with the starting time of soil erosion; specifically, the starting time was the 44th min., 66th min. and 74th min. Under the light rain test condition, the slope erosion started after the increase of the water content of the surface layer reached a high value for about 3 min. Under moderate rainfall test conditions, the rate of increase in soil water content peaked once, i.e., the rate of increase in soil water content reached 3.7%/min. at the 12th min., and the slope runoff and soil erosion started at the 13th min., which means the soil erosion started on the slope 1 min. after the surface water content reached its peak value. Under the conditions of the first heavy rain test, there was a peak in the rate of increase in soil water content. The rate of increase in soil water content reached 3.5%/min. at the 11th min., the slope runoff started at the 13th min., and the soil erosion started at the 17th min. Under the conditions of the first heavy rain test, the increase in surface soil water content reached its peak and slope runoff began about 1 min. later, followed by soil erosion about 6 min. later. Under the second heavy rainfall test condition, the rate of increase of soil water content peaked once, i.e. at the 8th min., the rate of increase of soil water content reached 1.5%/min., the slope runoff started at the 9th min., and the soil erosion started at the 10^th^ min. i.e. under the second heavy rainfall test condition, the increase of water content in the surface layer reached the peak for about 2 min. and then erosion started on the slope surface.

The above analysis shows that, when other conditions are constant, the soil water content and the change rate of water content will increase with the increase of rainfall, and the starting time of slope erosion is closely related to the rate of increase of the water content in the surface soil.

The aforementioned in-situ test results have provided a quantitative analysis of soil erosion in the Dabie Mountains area under various rainfall conditions. These findings have shed light on the key influencing factors of soil erosion within the study area. Furthermore, from the perspective of rainfall infiltration mechanisms into the soil, an innovative proposal has been put forward regarding the impacts of initial water content and its rate of change on vegetation, soil, and water conservation. This proposal has laid a theoretical foundation for the implementation of soil and water conservation measures in the study area. However, there is potential for further refinement of this experimental study. If additional experimental sites are available, incorporating sites without vegetation cover could allow for a quantitative assessment of the soil and water conservation effects associated with different vegetation types. Moreover, if the experimental water supply is adequate, it would be beneficial to superimpose different rainfall types to conduct rainfall experiments, thereby investigating the effects of compound rainfall on soil erosion.

## 5. Discussion

### 5.1 Key factors influencing soil erosion

#### 5.1.1 Effects of changes in soil water content and its derived indicators on soil erosion.

Soil erosion is a complex process of multiple factors, with rainfall being a paramount contributor [48]. Soil erosion initiates when rainfall droplets impact the soil, disrupting the cohesion between soil particles. Additionally, as rainwater infiltrates the soil, its water content rises, reducing soil stability and weakening its structural integrity. This makes soil particles susceptible to detachment, which, when transported by surface runoff, exacerbates soil erosion [49]. Previous studies usually used soil saturated water content as a discriminator for the occurrence of significant soil erosion. In fact, in most areas where significant erosion occurs, the soil is still unsaturated [50,51]. This study also proves this point of view through the rainfall in-situ test: when significant soil erosion occurs in the three test areas, the soil water content at all depths show an increasing trend, but the soil has not reached saturation. Through analyzing the response of slope erosion to the rate of change of soil water content, it was learnt that slope erosion mainly occurred when rainfall caused an increase in the rate of change in soil water content in the surface layer. as rainfall infiltrates and approaches soil water content saturation, a water film forms on the soil surface layer, slowing down the impact of raindrops; meanwhile, sediment production significantly decreased, indicating that significant erosion occurs during the period of increasing soil water content [52]. The findings of this study align with those reported in previous literature. Consequently, the rate of change in soil water content proposed in this study can serve as a reference indicator for significant soil erosion. From the above discussion, it is evident that the crucial aspect of rainfall-induced soil erosion lies in the increase in soil water content due to rainfall infiltration. The management of rainfall infiltration is a pivotal scientific issue. Numerous studies have confirmed that the initial soil water content prior to rainfall is a significant factor influencing rainfall infiltration [53]. Specifically, a higher initial soil water content results in a lower initial infiltration rate, though its impact on the stabilized infiltration rate is negligible. However, under rainfall infiltration conditions, the shear strength of the soil is affected by variations in initial soil water content, leading to differing bonding forces among soil particles and varying stability of soil aggregates [54]. Castillo et al [55]. concluded that initial soil moisture content is a crucial control factor for runoff generation in semi-arid environments, a finding consistent with this study. Therefore, maintaining an optimal level of soil moisture is a vital strategy for preventing soil erosion during the rainy season in the Dabie Mountains area.

#### 5.1.2 Impact of land-use change on soil erosion.

The primary emphasis of this thesis study is the erosion phenomenon observed in the soils of the Dabie Mountains area following the transition from forested land to the cultivation of cash crops. These changes are induced by alterations in factors such as micro-geomorphology, soil physical properties, and surface vegetation cover, which subsequently influence regional soil erosion dynamics. Consequently, these dynamics modify the mechanism of yield-sink formation within the affected area, further influencing soil erosion [56]. Irrational land-use practices, including overgrazing, clearing of steep slopes, indiscriminate deforestation, and irrational changes in cropping patterns, contribute to or even exacerbate soil erosion [57,58]. Zare et al [59]. investigated the influence of various land-use change scenarios on soil erosion in northern Iran using the RUSLE model. Anache et al. [60] compiled field data on runoff and soil erosion in Brazil, revealing that the relationship among annual precipitation, annual runoff, and soil erosion in different regions is primarily influenced by the spatial and temporal patterns of land-use types and vegetation cover. Researchers further analyzed the impact of climate change on runoff and erosion in various land-use types in Europe and the Mediterranean region by compiling data from the subcontinent. They found that soil erosion was more severe in artificial vegetation types compared to natural vegetation types [61]. The influence of land-use change on soil erosion is multifaceted. On one hand, land-use change affects precipitation, evaporation, and runoff in numerous ways, thereby impacting the redistribution of regional water resources and the entire water cycle. On the other hand, land-use changes can alter the regional water cycle by modifying water-heat transport, albedo, and net radiation. When the water cycle is altered, the shear strength of the soil also changes, which in turn affects the severity of soil erosion [62]. In recent years, experts and scholars have calculated the soil erosion modulus for different land-use types, such as forest land, grassland and arable land, and quantitatively estimated the degree of soil erosion in the study area. The results showed significant differences based on land-use types [63–65], which also corroborate the findings of this thesis. However, this does not imply that the soil and water conservation effect of forest land in the same area is inherently superior to that of grassland or cropland, and other factors such as vegetation type and vegetation cover should also be considered.

### 5.2 Mechanisms of plant measures in soil and water conservation

The issue of soil erosion resulting from vegetation destruction is among the most prevalent ecological challenges [66,67]. Vegetation serves as an effective barrier against the erosive forces of wind and water on soil [68]. This manuscript delves into the role of vegetation in controlling water erosion. Vegetation mitigates the volume and velocity of surface runoff by fostering a robust soil structure, enhancing soil porosity, and improving water permeability [69]. These findings align with the experimental results presented in this paper. Specifically, under consistent conditions, an increase in vegetation cover corresponds to an elevation in soil water content. Notably, vegetation enhances soil permeability primarily through the plant’s root system. Otuaro et al. [70]. argue that the root system’s role in soil penetration is evident in its capacity to bind soil particles together while also disaggregating compacted soil mass and synthesizing humus through decomposition and transformation processes, thereby granting the soil a favorable aggregation structure and pore space. Under identical conditions, a more developed vegetation root system decreases the likelihood of soil erosion. This insight offers a foundational principle for selecting plants to manage severely eroded areas effectively.

Furthermore, the vegetation canopy and litter layer can diminish raindrop splash erosion and intercept a portion of precipitation, thereby reducing surface runoff volume and safeguarding the surface soil from erosion. Numerous scholars have conducted corresponding research in this area. Their studies have examined the impact of various vegetation covers on soil erosion, primarily through remote sensing monitoring and indoor simulation experiments [29,71,72]. Ma et al. [73]. simulated the characteristics of water and sediment transport on slopes with different land-use scenarios under varying rainfall conditions in the Loess Plateau region. They discovered that shrubs with a cover exceeding 20% experience erosion in the middle and lower sections of slopes, whereas increasing vegetation cover to 30% and above significantly slows down soil erosion on these slopes. This result echoes the findings of this paper. In summary, vegetation generally regulates the rate and path of both surface and subsurface water flow on most slopes, thereby mitigating soil erosion caused by rainfall.

## 6. Conclusions

In this study, the influence of internal and external factors, such as rainfall and vegetation, on soil erosion on the slopes of Dabie Mountain was investigated by conducting in situ experiments. The main results and conclusions are as follows:

(1) The impact of rainfall on soil erosion is mainly reflected in the increase in the rate of change of the water content in the top soil caused by rainfall, and the rapid increase in the water content in the top soil makes the soil stability decrease dramatically, leading to the occurrence of soil erosion on the slope. Therefore, the rate of change of water content in the top soil can be used as a key discriminative basis for the occurrence of soil erosion on slopes.
(2) In Dabie Mountains area, when all other conditions remain constant, if the rainfall exceeds 40 mm or more, the initial soil moisture content decreases by more than 2%. Consequently, the volume of runoff from the slope surface increases by more than 0.4 cubic meters, and the amount of soil loss rises by over 0.13 kg. Therefore, the initial water content of the soil is an important factor influencing the occurrence of soil erosion on slopes. Within the permeation range of rainfall, if the soil experiences drought conditions, the initial soil water content decreases, and then strong rainfall occurs, it is easy to make the soil water content increase rapidly, drastically reducing the stability of the soil, resulting in soil erosion on slopes, and significantly increasing the cumulative amount of soil erosion.
(3) In the Dabie Mountains, there are a large number of slopes where forest lands have been converted to tea and other cash crops. When other conditions remain constant, rainfall measuring 20 mm or more can reduce soil loss by over 80% when compared to tea fields with a vegetation cover of only 20%. Consequently, tea planting can exhibit a substantial soil-stabilizing effect, provided that the vegetation cover is at least 40%. It is noteworthy that vegetation’s effect on soil and water conservation also hinges on factors such as vegetation type, age, morphology, canopy height, root depth, and root shape. The influence of these indicator factors on soil and water conservation will be further explored in future research.

## Supporting information

S1 Fig Site plan of indoor rainfall intensity uniformity test. (TIF)

S2 Fig Sketch of rainfall uniformity test layout. (TIF)

S3 Fig Flow chart of the rainfall in-situ test. (TIF)
