# Supplementary figures and images for: Experimental study on in-situ simulation of rainfall-induced soil erosion in forest lands converted to cash crop areas in Dabie Mountains
Source: PLoS One. 2025 Feb 7;20(2):e0317889. doi: 10.1371/journal.pone.0317889 (PMC11805400; doi:10.1371/journal.pone.0317889)

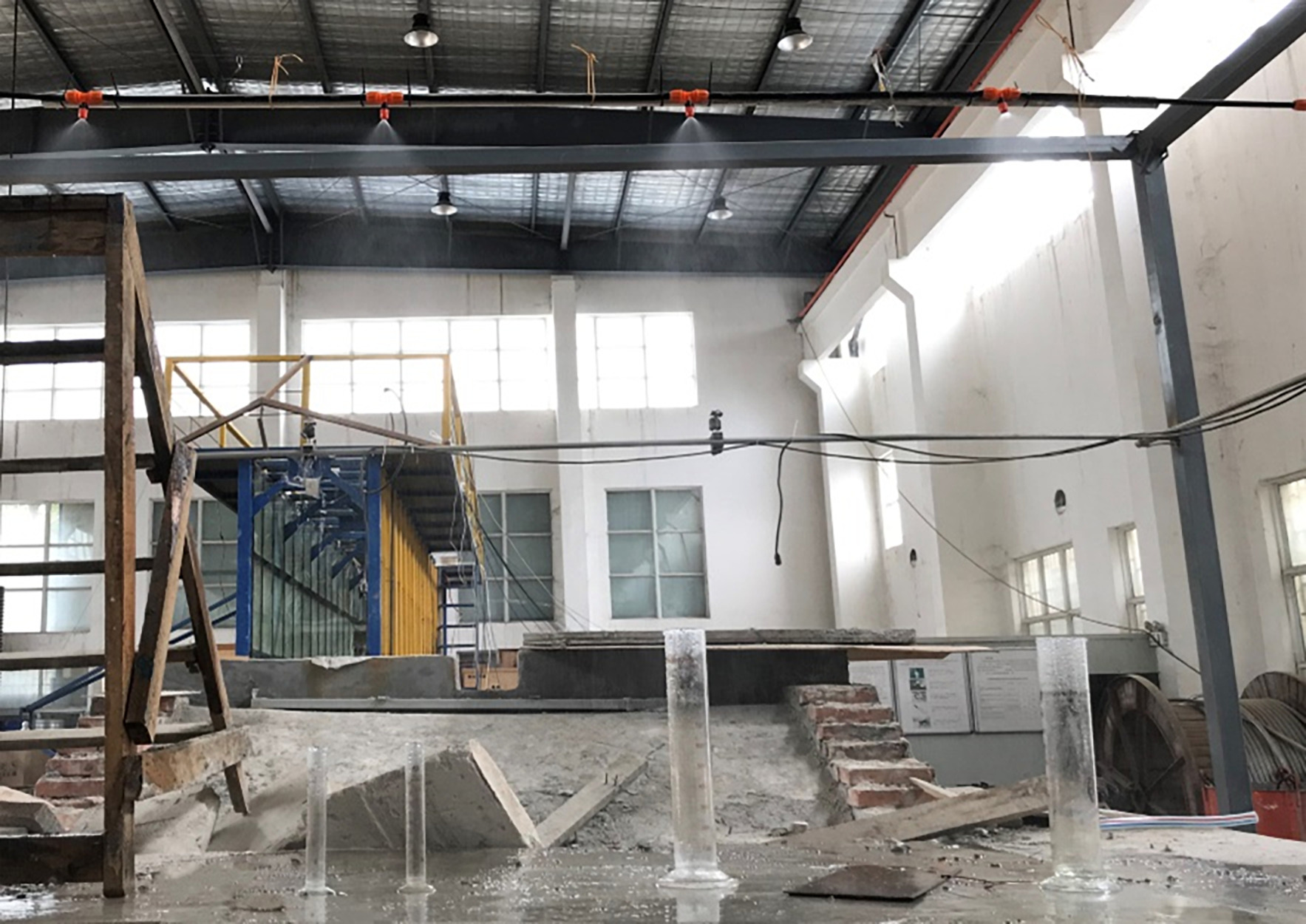

Supplement: S1 Fig — (TIF) [file pone.0317889.s001.tif]

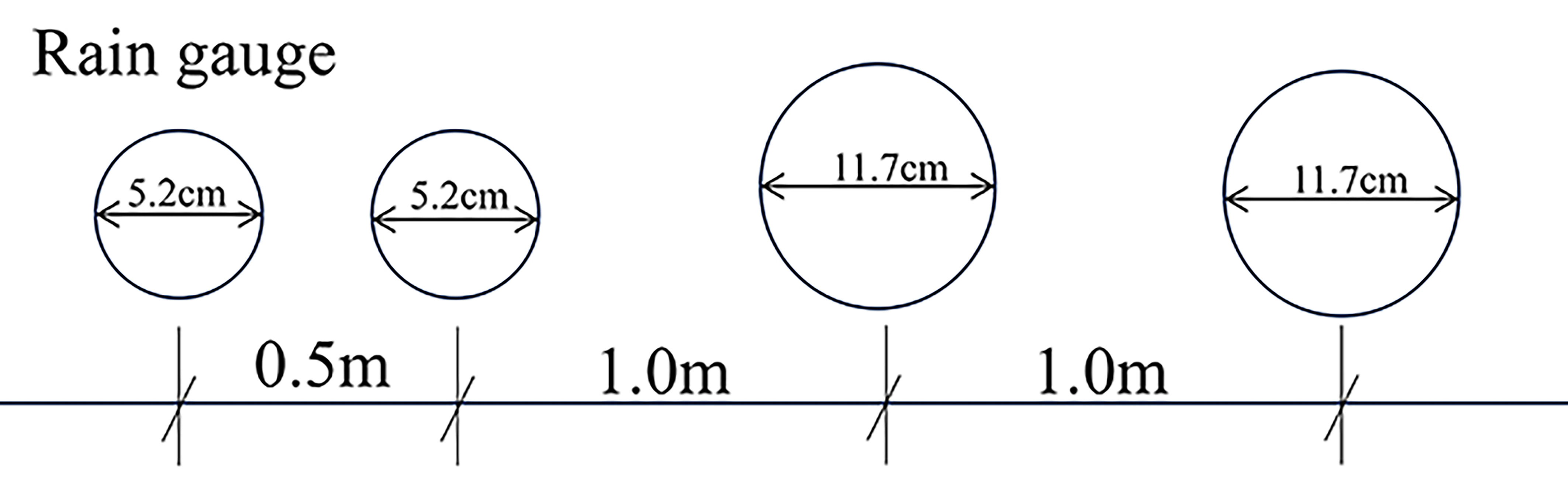

Supplement: S2 Fig — (TIF) [file pone.0317889.s002.tif]

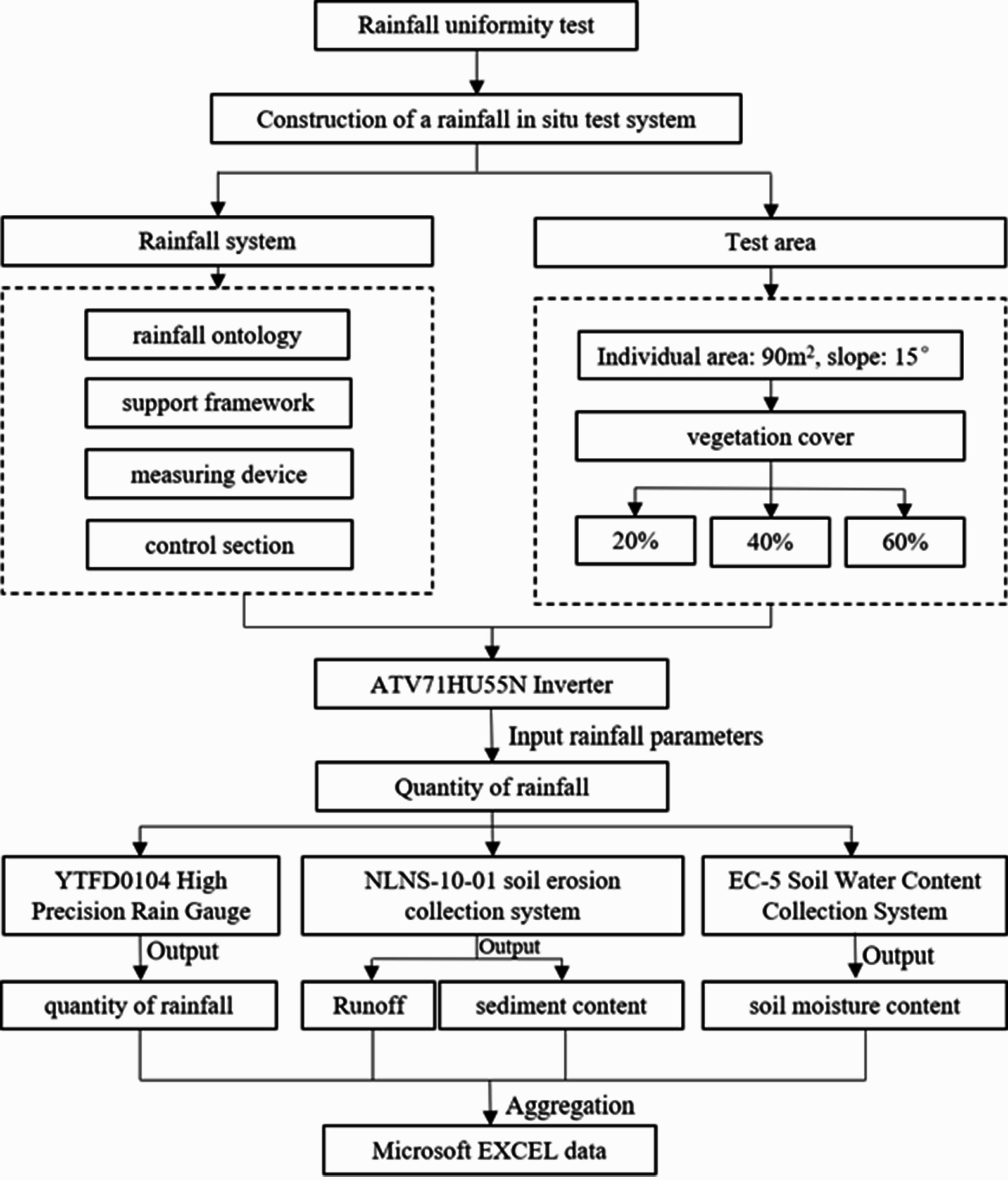

Supplement: S3 Fig — (TIF) [file pone.0317889.s003.tif]
